# Metabolic Syndrome and Breast Cancer Risk: A Case-Cohort Study Nested in a Multicentre Italian Cohort

**DOI:** 10.1371/journal.pone.0128891

**Published:** 2015-06-01

**Authors:** Claudia Agnoli, Sara Grioni, Sabina Sieri, Carlotta Sacerdote, Fulvio Ricceri, Rosario Tumino, Graziella Frasca, Valeria Pala, Amalia Mattiello, Paolo Chiodini, Licia Iacoviello, Amalia De Curtis, Salvatore Panico, Vittorio Krogh

**Affiliations:** 1 Epidemiology and Prevention Unit, Fondazione IRCCS - Istituto Nazionale dei Tumori, Milan, Italy; 2 Human Genetics Foundation, Turin, Italy; 3 Piemonte Centre for Cancer Prevention, Turin, Italy; 4 Cancer Registry, ASP 7 Ragusa, Italy; 5 Department of Clinical and Experimental Medicine, University of Naples Federico II, Naples, Italy; 6 Department of Mental and Physical Health and Preventive Medicine, Second University of Naples, Naples, Italy; 7 Laboratory of Molecular and Nutritional Epidemiology, Department of Epidemiology and prevention, IRCCS Istituto Neurologico Mediterraneo Neuromed, Pozzilli (IS), Italy; ISPO, ITALY

## Abstract

**Background:**

Metabolic syndrome (defined as at least three among abdominal obesity, high blood triglycerides, low high-density lipoprotein cholesterol, high blood glucose, and high blood pressure) is emerging as a risk factor for breast cancer; however few studies – most confined to postmenopausal women – have investigated associations between breast cancer risk and metabolic syndrome. The purpose of this study was to examine the association between metabolic syndrome and its components, and risk of breast cancer in postmenopausal and premenopausal women.

**Methods:**

We performed a case-cohort study on 22,494 women recruited in 1993-1998 to four Italian centres (Turin, Varese, Naples, Ragusa) of the European Prospective Investigation into Cancer and Nutrition (EPIC) and followed-up for up to 15 years. A random subcohort of 565 women was obtained and 593 breast cancer cases were diagnosed. Hazard ratios (HR) with 95% confidence intervals (CI), adjusted for potential confounders, were estimated by Prentice-weighted Cox proportional hazards models.

**Results:**

Presence of metabolic syndrome was associated with significantly increased breast cancer risk in all women (HR 1.52, 95%CI 1.14-2.02). When the analyses were repeated separately for menopausal status, the association was limited to postmenopausal women (HR 1.80, 95%CI 1.22-2.65) and absent in premenopausal women (HR 0.71, 95%CI 0.43-1.16); P for interaction between metabolic syndrome and menopausal status was 0.001. Of metabolic syndrome components, only high blood glucose was significantly associated with increased breast cancer risk in all women (HR 1.47, 95%CI 1.13-1.91) and postmenopausal women (HR 1.89, 95%CI 1.29-2.77), but not premenopausal women (HR 0.80, 95%CI 0.52-1.22; P interaction=0.004).

**Conclusions:**

These findings support previous data indicating that metabolic syndrome is an important risk factor for breast cancer in postmenopausal women, but not in premenopausal women, and suggest that prevention of metabolic syndrome through lifestyle changes could confer protection against breast cancer.

## Introduction

Breast cancer is the most common cancer in women, and second most common cancer; it is the first cause of cancer death in women and fifth most important cause of cancer death [1]. Although genetic factors play a role in the development of breast cancer, the main risk factors are hormonal, reproductive and lifestyle [2].

Metabolic syndrome is a cluster of metabolic abnormalities that occur in persons with impaired insulin sensitivity [3]. It is defined by the presence of at least three of the following: abdominal obesity, high blood triglycerides, low high-density lipoprotein (HDL) cholesterol, high blood pressure, and high fasting glucose [4]. A recent systematic review on women aged 18 years or older found a modest positive association between metabolic syndrome and breast cancer [5]. Another systematic review based on nine observational studies found moderately increased risk of breast cancer in postmenopausal women with metabolic syndrome [6]. Of the thirteen studies included in the two reviews [7–19], only three considered premenopausal women separately [7–9], with mixed results. Metabolic syndrome could influence breast cancer risk through effects on interrelated signalling pathways involving insulin, estrogens, growth factors, and cytokines [20, 21].

Since breast cancer is conspicuously a hormone-related disease, risk factors could have different effects depending on menopausal status [22]; however effects of premenopausal vs. postmenopausal status on the relationship between metabolic syndrome and breast cancer risk have not been extensively investigated.

The aim of this case-cohort study was to investigate the association of metabolic syndrome and its components with breast cancer risk. We investigated all women and postmenopausal and premenopausal women separately.

## Materials and Methods

### Study population and data collection

Our case-cohort study to investigate breast cancer risk in women with metabolic syndrome was nested in the population of four Italian centres of the European Prospective Investigation into Cancer and Nutrition (EPIC), and formed part of a wider case-cohort design study to investigate three other outcomes (myocardial infarction, stroke, and colorectal cancer). The study protocol was approved by the ethics committee of the Human Genetics Foundation (Turin, Italy). The study complies with the Helsinki declaration, and participants gave informed consent to use clinical data for research.

The cohort consisted of 22,494 women recruited prospectively in 1993–1998 to the four EPIC-Italy centres of Varese and Turin (Northern Italy), Naples and Ragusa (Southern Italy). At baseline, after participants had given written informed consent, detailed information was collected on lifestyle using a standardized lifestyle questionnaire, and on diet over the previous year using food frequency questionnaires specifically developed to capture local dietary habits [23]. Also at baseline, weight, height, and blood pressure were measured [24] and a 30 ml fasting blood sample was taken, all according to standardized procedures. The blood samples were divided into 0.5 ml aliquots of plasma, serum, red blood cells, and buffy coat, on the day of collection, and stored in liquid nitrogen at -196°C [25].

### Cohort definition, identification of cases, and follow-up

A centre-stratified random sample of 565 participants was obtained from the 22,494 women, to serve as subcohort, which included 16 women who developed breast cancer during follow-up.

In Varese, Turin, and Ragusa, incident breast cancers were identified by linkage to the databases of regional cancer registries. In Naples, incident cases were identified through linkage to the regional archive of hospital discharges, and by direct telephone contact where necessary.

Cancers were primary incident cases (invasive and in situ) identified by codes C50.0–50.9 of the International Classification of Diseases (10th Revision). A total of 618 cases was identified.

Women were followed-up from study entry until the first cancer diagnosis (except non-melanoma skin cancer), death, emigration, or end of follow-up, whichever occurred first. End of follow-up varied with centre: December 31, 2006 for Varese and Naples; December 31, 2008 for Turin and Ragusa.

After excluding women with unavailable plasma samples or missing lifestyle information, the sample on which we performed statistical analyses therefore consisted of 1133 participants: 555 in the randomly selected subcohort and 593 cases (15 in the subcohort).

### Analysis of plasma samples

Triglycerides, HDL cholesterol and glucose were measured in plasma samples using commercial colorimetric enzyme kits (Instrumentation Laboratory, Milan, Italy) and an automatic analyser (IL 350, Instrumentation Laboratory). Quality control was checked using commercial (high and low) laboratory standards and an in-house plasma pool. Coefficients of variation (CV) for high level external standards were 5.0% for triglycerides, 6.1% for HDL cholesterol, and 5.0% for glucose. CVs for low level external standards were 7.9% for triglycerides, 7.0% for HDL cholesterol, and 7.6% for glucose. CVs for the in-house plasma pool were 3.5% for triglycerides, 5.3% for HDL cholesterol and 3.8% for glucose.

To render the results in plasma samples comparable with literature data on serum samples, the following conversion factors were applied: 1.338 for triglycerides, 1.344 for HDL cholesterol, 1.181 for glucose. These factors were determined in the laboratory by comparison of analyte concentrations in plasma and serum from 222 persons. For all analyses, laboratory staff were blind to the case-control status of samples.

### Definition of metabolic syndrome

Most definitions of metabolic syndrome require the presence of at least three of the following five components: abdominal obesity, high triglycerides, low HDL cholesterol, high blood glucose, and high blood pressure. Definitions differ mainly as regards the cut-offs used for each component. We used the latest harmonized definition [4] of metabolic syndrome (slightly modified) that requires the presence of three or more of the following: waist circumference ≥80 cm; triglycerides ≥150 mg/dl; HDL cholesterol <50 mg/dl; fasting glucose ≥100 mg/dl or diabetes treatment (self-reported in our study); systolic blood pressure ≥130 mm Hg, and diastolic blood pressure ≥85 mm Hg or antihypertensive drug treatment. The harmonized definition includes drug treatment for high triglycerides and low HDL cholesterol, but we ignored these as drug information was not available.

### Statistical analysis

Baseline characteristics of cohort members, according to presence/absence of metabolic syndrome, were summarized as means and standard deviations (continuous variables) or frequencies (categorical variables). Prentice-weighted Cox proportional hazards models, with age as underlying time variable, were used to assess associations of metabolic syndrome and its components with breast cancer risk [26]. We compared women with and without metabolic syndrome and women categorized as: 0 metabolic syndrome components, 1–2 components, and 3–5 components. We also compared women with and without each individual component. We ran unadjusted models and fully-adjusted models, with the following covariates: menopausal status (premenopausal, postmenopausal, perimenopausal), parity (nulliparous, 1–2 full-term pregnancies, >2 full term-pregnancies), age at menarche (<15 years, ≥15 years), smoking status (never, former, current), total physical activity (inactive, moderately inactive, moderately active, and active), education (≤8 years, >8 years), and alcohol consumption (≤0.1 g/d, >0.1 to ≤12 g/d, >12 to ≤24 g/d, >24 g/d). Further models were also adjusted for BMI. All models were stratified by centre and age (5-year classes). The significance of linear trends across categories of metabolic syndrome components was tested by treating each category as a continuous variable in the Cox models. We ran models for the whole cohort and for premenopausal and postmenopausal women. As a sensitivity analysis, we ran models excluding women in treatment for diabetes.

P values for interaction of exposure variables with menopausal status were calculated adding to the models product terms for exposure (presence/absence) and menopausal status. The analyses were performed with Stata version 11.2 (College Station, TX, USA).

## Results

Table 1 shows baseline characteristics of the subcohort divided into those with and without metabolic syndrome. Compared to women without metabolic syndrome (n = 406), those with metabolic syndrome (n = 149) were older and had higher body mass index (BMI). Those with metabolic syndrome were also more likely to be postmenopausal, less educated, less physically active, smoked less, had later age at menarche, and more than 2 full-term pregnancies.

**Table 1:** Baseline characteristics of the subcohort according to presence or absence of metabolic syndrome.

*Characteristic*	*With metabolic syndrome (n = 149)*	*Without metabolic syndrome (n = 406)*
	Mean ± SD	
Age, years	53.53 ± 7.58	49.34 ± 8.04
Body mass index, kg/m^2^	29.61 ± 4.54	24.86 ± 3.69
	N (%)	
Centre:		
- Turin	68 (45.6)	174 (42.9)
- Varese	18 (12.1)	61 (15.0)
- Naples	24 (16.1)	79 (19.5)
- Ragusa	39 (26.2)	92 (22.6)
Menopausal status:		
- Postmenopausal	86 (57.7)	164 (40.4)
- Premenopausal	56 (37.6)	201 (49.5)
- Perimenopausal	7 (4.7)	41 (10.1)
Age at menarche:		
- <15 years	129 (86.6)	378 (93.1)
- ≥15 years	20 (13.4)	28 (6.9)
Parity:		
- Nulliparous	9 (6.1)	49 (12.1)
- 1–2 full-term pregnancies	86 (57.7)	268 (66.0)
- >2 full-term pregnancies	54 (36.2)	89 (21.9)
Smoking status:		
- Current smoker	20 (13.4)	115 (28.3)
- Former smoker	26 (17.5)	69 (17.0)
- Never smoker	103 (69.1)	222 (54.7)
Education:		
- ≤8 years	101 (67.8)	193 (47.5)
- >8 years	48 (32.2)	213 (52.5)
Alcohol consumption:		
- ≤0.1 g/d	39 (26.2)	89 (21.9)
- >0.1–12 g/d	79 (53.0)	226 (55.7)
- >12–24 g/d	17 (11.4)	58 (14.3)
- >24 g/d	14 (9.4)	33 (8.1)
Total physical activity:		
- Inactive	72 (48.3)	146 (36.0)
- Moderately inactive	53 (35.6)	169 (41.6)
- Moderately active	14 (9.4)	49 (12.1)
- Active	10 (6.7)	42 (10.3)

Table 2 shows hazard ratios (HRs) with 95% confidence intervals (CIs) for developing breast cancer according to presence versus absence of metabolic syndrome and according to number of metabolic syndrome components, in all study women, and in postmenopausal and premenopausal women separately. Considering the fully-adjusted models (excluding BMI) only, for all study women, presence of metabolic syndrome was associated with significantly greater breast cancer risk than absence of metabolic syndrome (HR 1.52, 95%CI 1.14–2.02). When number of metabolic syndrome components was considered, the highest number category (≥3 components) was associated with significantly greater breast cancer risk than reference (0 components) (HR 1.91, 95%CI 1.28–2.84), with a significant linear trend (P = 0.001). For postmenopausal women, the increase in risk was greater than for all women, both for presence versus absence of metabolic syndrome (HR 1.80, 95%CI 1.22–2.65) and for increasing number of metabolic syndrome components (HR 3.12, 95%CI 1.43–6.79 for ≥3 components versus 0 components, P trend = 0.001).

**Table 2:** Hazard ratios (HRs) for developing breast cancer in relation to metabolic syndrome and number of metabolic syndrome components.

	Cases/Controls	HR^1^	HR^2^
***Whole cohort***			
Absence of metabolic syndrome (<3 components)	387/406	1	1
Presence of metabolic syndrome (≥3 components)	206/149	**1.50 (1.14–1.97)**	**1.52 (1.14–2.02)**
P interaction^3^		**0.001**	**0.001**
Number of components (3 categories)			
0	86/115	1	1
1–2	301/291	1.32 (0.94–1.85)	1.33 (0.94–1.89)
≥3	206/149	**1.85 (1.28–2.69)**	**1.91 (1.28–2.84)**
P trend		**0.001**	**0.001**
***Postmenopausal women***			
Absence of metabolic syndrome (<3 components)	153/164	1	1
Presence of metabolic syndrome (≥3 components)	133/86	**1.85 (1.28–2.69)**	**1.80 (1.22–2.65)**
Number of components (3 categories)			
0	17/30	1	1
1–2	136/134	1.64 (0.82–3.27)	1.89 (0.89–4.01)
≥3	133/86	**2.80 (1.38–5.71)**	**3.12 (1.43–6.79)**
P trend		**0.001**	**0.001**
***Premenopausal women***			
Absence of metabolic syndrome (<3 components)	201/201	1	1
Presence of metabolic syndrome (≥3 components)	57/56	0.72 (0.45–1.15)	0.71 (0.43–1.16)
Number of components (3 categories)			
0	62/71	1	1
1–2	139/130	1.11 (0.73–1.68)	1.12 (0.72–1.74)
≥3	57/56	0.77 (0.45–1.34)	0.77 (0.43–1.40)
P trend		0.445	0.478

^1^Stratified by age (5-year classes) and centre.

^2^Adjusted for menopausal status (whole cohort model only), number of full-term pregnancies, age at menarche, smoking status, education, physical activity, and alcohol intake; stratified by age (5-year classes) and centre.

^3^P for interaction between metabolic syndrome and menopausal status.

For premenopausal women, presence of metabolic syndrome was associated with a non-significantly lower breast cancer risk than absence of metabolic syndrome (HR 0.71, 95%CI 0.43–1.16). A significant interaction (P = 0.001) between metabolic syndrome and menopausal status was found. Breast cancer risk did not vary significantly with number of metabolic syndrome components.

When BMI was added to the models, results were closely similar to those of the fully-adjusted models (data not shown). The results of model run after excluding women who self-reported diabetes, were also closely similar to the results of the models (Table 2) that included all women (data not shown).

When presence versus absence of individual metabolic syndrome components was analysed (Table 3), only high fasting glucose was significantly associated with increased breast cancer risk in the fully-adjusted models, both in all women (HR 1.47, 95%CI 1.13–1.91) and in postmenopausal women (HR 1.89, 95%CI 1.29–2.77). In premenopausal women, we found a non-significantly lower risk associated with high fasting glucose (HR 0.80, 95%CI 0.52–1.22). The interaction between fasting glucose and menopausal status was significant (P interaction = 0.004). The interaction between blood pressure and menopausal status was also significant (P interaction = 0.023), although no significant association between this component with breast cancer risk was found, either in the analysis on all women or the analyses according to menopausal status.

**Table 3:** Hazard ratios (HRs) for developing breast cancer in relation to metabolic syndrome components.

	Cases/Controls	HR^1^	HR^2^
***Whole cohort***			
High waist circumference			
No	254/249	1	1
Yes	339/306	1.11 (0.86–1.42)	1.07 (0.82–1.39)
P interaction^3^		0.101	0.130
High triglycerides			
No	439/430	1	1
Yes	154/125	1.24 (0.93–1.66)	1.22 (0.90–1.64)
P interaction^3^		0.355	0.530
Low HDL cholesterol			
No	494/464	1	1
Yes	99/91	1.27 (0.90–1.80)	1.25 (0.88–1.79)
P interaction^3^		0.503	0.612
High blood pressure			
No	249/256	1	1
Yes	344/299	1.22 (0.94–1.58)	1.24 (0.95–1.63)
P interaction^3^		**0.031**	**0.023**
High fasting glucose			
No	382/414	1	1
Yes	211/141	**1.42 (1.10–1.84)**	**1.47 (1.13–1.91)**
P interaction^3^		**0.001**	**0.004**
***Postmenopausal women***			
High waist circumference			
No	96/88	1	1
Yes	190/162	1.12 (0.75–1.63)	1.04 (0.69–1.57)
High triglycerides			
No	195/177	1	1
Yes	91/73	1.25 (0.84–1.86)	1.21 (0.80–1.83)
Low HDL cholesterol			
No	240/212	1	1
Yes	46/38	1.24 (0.75–2.07)	1.11 (0.64–1.93)
High blood pressure			
No	71/75	1	1
Yes	215/175	1.46 (0.96–2.22)	1.51 (0.96–2.39)
High fasting glucose			
No	161/182	1	1
Yes	125/68	**1.95 (1.34–2.85)**	**1.89 (1.29–2.77)**
***Premenopausal women***			
High waist circumference			
No	135/132	1	1
Yes	123/125	0.80 (0.54–1.17)	0.77 (0.51–1.16)
High triglycerides			
No	210/214	1	1
Yes	48/43	1.04 (0.65–1.66)	1.07 (0.66–1.75)
Low HDL cholesterol			
No	219/212	1	1
Yes	39/45	1.06 (0.64–1.76)	0.99 (0.58–1.67)
High blood pressure			
No	154/152	1	1
Yes	104/105	0.83 (0.57–1.21)	0.88 (0.60–1.31)
High fasting glucose			
No	189/193	1	1
Yes	69/64	0.75 (0.50–1.12)	0.80 (0.52–1.22)

^1^Stratified by age (5-year classes) and centre.

^2^Adjusted for menopausal status (whole cohort model only), number of full-term pregnancies, age at menarche, smoking status, education, physical activity, and alcohol intake; stratified by age (5-year classes) and centre.

^3^P for interaction between metabolic syndrome components and menopausal status.

## Discussion

In this case-cohort study, we have found that presence of metabolic syndrome was associated with increased breast cancer risk in all women, but the risk was greater in postmenopausal women, while among premenopausal women, a non-significantly lower risk of developing breast cancer was found. Considering the individual components of metabolic syndrome, high fasting glucose was significantly associated with increased breast cancer risk in all women and in postmenopausal women, while in premenopausal women, the association tended in the opposite direction (not significant). High blood pressure also had opposing non-significant effects in postmenopausal (increased risk) and premenopausal women (decreased risk).

Our finding of increased breast cancer risk in women with metabolic syndrome is in line with two recently published meta-analyses. The first, based on nine prospective studies on postmenopausal women, found that presence of metabolic syndrome was associated with a significant 52% increase in breast cancer risk [6]. The other, which included both postmenopausal and premenopausal women, found a significant 47% increase in breast cancer risk; the study did not analyse risk according to menopausal status [5].

Few studies have assessed metabolic syndrome and breast cancer risk in premenopausal women, and results have been contrasting. The Me-Can project [7], which pooled six cohorts from Austria, Sweden and Norway, and included 1046 breast cancer cases less than 50 years at the end of follow-up, found that risk of breast cancer was significantly lower among those with metabolic syndrome. A prospective cohort study on 34 black premenopausal US women with metabolic syndrome who subsequently developed breast cancer [8] found no significant association between metabolic syndrome and breast cancer risk. Finally, a Uruguayan hospital-based case-control study on 253 premenopausal women with breast cancer diagnosed between 2004–2009 and 497 frequency-matched healthy controls [9], found that risk was non-significantly greater in women with three or more metabolic syndrome components versus those with no components. The discrepant findings of these three studies on premenopausal women could be due to differences in study design (cohort vs. case-control), definition of metabolic syndrome, method of adjusting for confounding variables, and definition of menopausal status. As regards the latter, studies [8] and [9] determined menopausal status at recruitment, while study [7] considered age at the end of follow-up.

Several mechanisms have been proposed to explain the association between metabolic syndrome and postmenopausal breast cancer risk. The pathophysiological state that underlies metabolic syndrome is insulin resistance, which can increase breast cancer risk [27] via several mechanisms that could act additively or synergistically. Hyperinsulinemia—consequence of insulin resistance—increases the bioavailability of insulin-like growth factor (IGF)-1 by inhibiting liver production of IGF-binding proteins [28, 29]. Hyperinsulinemia also stimulates the expression of growth hormone receptor (GHR) in liver [30] to possibly increase liver IGF-1 production by enhanced GHR signalling there [30]. Both IGF-1 and insulin are mitogenic and anti-apoptotic for human breast epithelium and also human breast cancer cells [31–33]; human breast cancer cells often overexpress IGF-1 receptor and insulin receptor [34].

Insulin may also enhance breast cancer risk, especially in postmenopausal women, by increasing levels of estrogens and androgens. Estrogens have a causal role in breast cancer [35], especially in postmenopausal women [36], while high levels of total and free testosterone have also been associated with increased breast cancer risk in postmenopausal women [37]. Insulin stimulates the ovarian production of androgens [38], whose aromatization in peripheral tissues is the main source of estrogen after the menopause [39]; insulin also upregulates aromatase activity [40]. In addition, insulin inhibits liver production of sex hormone binding globulin (SHBG), thereby increasing the bioavailability of both estrogens and androgens [41].

Metabolic syndrome is characterized by high levels of inflammatory cytokines [42] and leptin [43] which can promote breast cancer cell growth through various mechanisms [44, 45], and decreased levels of adiponectin [46], resulting in lack of downregulation of tumour cell proliferation and inhibition of apoptosis [45].

Finally, Beaulieu et al. [47] have suggested that metabolic syndrome exerts a effect on the breast cancer microenvironment to support cancer invasion. They propose that plasminogen activator inhibitor-1 (PAI-1) is involved in this effect via a ‘PAI-1 cycle’, in which metabolic syndrome upregulates PAI-1 expression to promote angiogenesis, tumour cell migration and procoagulant microparticle formation from endothelial cells, which in turn generate thrombin and further propagate PAI-1 synthesis [47]. Our previous study on the same cases and subcohort as used in the present study, found that high PAI-1 was significantly associated with increased risk of breast cancer, colorectal cancer and ischaemic stroke [48].

Our finding of a non-significant inverse association between metabolic syndrome and breast cancer risk in premenopausal women is less easy to explain. We tentatively suggest that insulin’s stimulatory effect on ovarian androgen synthesis may lead to the development of ovarian hyperandrogenism in susceptible women [49], which in turn may reduce breast cancer risk in premenopausal women by causing chronic anovulation with reduced ovarian production of estradiol and progesterone [50].

Strengths of our study are its prospective design, relatively large sample size, and availability of detailed information on lifestyle that made it possible to control for confounding effects.

A limitation is that we assessed variables at baseline only and do not know to what extent they may have changed subsequently. Nevertheless there is good reason to believe that blood chemistry remains relatively stable over time [51]. Furthermore, we cannot rule out confounding by factors we were unable to estimate (or estimated sub-optimally) in our questionnaires.

To conclude, we found that metabolic syndrome is a risk factor for postmenopausal breast cancer but not for premenopausal breast cancer. Of the individual components of the syndrome, elevated fasting glucose had the strongest association with risk in postmenopausal women. These findings suggest that prevention of metabolic syndrome by lifestyle changes might have an important protective effect against the development of breast cancer.
